# Profiling of Invasive Breast Carcinoma Circulating Tumour Cells—Are We Ready for the ‘Liquid’ Revolution?

**DOI:** 10.3390/cancers11020143

**Published:** 2019-01-25

**Authors:** Marcin Braun, Aleksandra Markiewicz, Radzisław Kordek, Rafał Sądej, Hanna Romańska

**Affiliations:** 1 Department of Pathology, Chair of Oncology, Medical University of Lodz, 92-213 Lodz, Poland; braunmarcin@gmail.com (M.B.); radzislaw.kordek@umed.lodz.pl (R.K.); 2 Department of Medical Biotechnology, Medical University of Gdansk, 80-211 Gdansk, Poland; rsadej@gumed.edu.pl

**Keywords:** invasive breast cancer, circulating tumour cells, predictive/prognostic multiparametric panels, gene signatures, single cells analysis

## Abstract

As dissemination through blood and lymph is the critical step of the metastatic cascade, circulating tumour cells (CTCs) have attracted wide attention as a potential surrogate marker to monitor progression into metastatic disease and response to therapy. In patients with invasive breast carcinoma (IBC), CTCs are being considered nowadays as a valid counterpart for the assessment of known prognostic and predictive factors. Molecular characterization of CTCs using protein detection, genomic and transcriptomic panels allows to depict IBC biology. Such molecular profiling of circulating cells with increased metastatic abilities appears to be essential, especially after tumour resection, as well as in advanced disseminated disease, when information crucial for identification of therapeutic targets becomes unobtainable from the primary site. If CTCs are truly representative of primary tumours and metastases, characterization of the molecular profile of this easily accessible ‘biopsy’ might be of prime importance for clinical practice in IBC patients. This review summarizes available data on feasibility and documented benefits of monitoring of essential IBC biological features in CTCs, with special reference to multifactorial proteomic, genomic, and transcriptomic panels of known prognostic or predictive value.

## 1. Introduction

Invasive breast carcinoma (IBC) is the most frequent and the deadliest cancer in women worldwide; however, identification of its predictive features and application of this knowledge in clinical practice have brought a huge progress in effective management of IBC patients [1,2,3,4]. The factors predicting disease course encompass both routinely assessed pathological (lymph node status, grading, tumour size and extent) and biological characteristics, predominantly the steroid hormone receptor status (Estrogen Receptor (ER), Progesterone Receptor (PR)), as well as human epidermal growth factor receptor 2 (HER2) expression/amplification status and Ki67 proliferation index [2]. 

**Figure 1:**
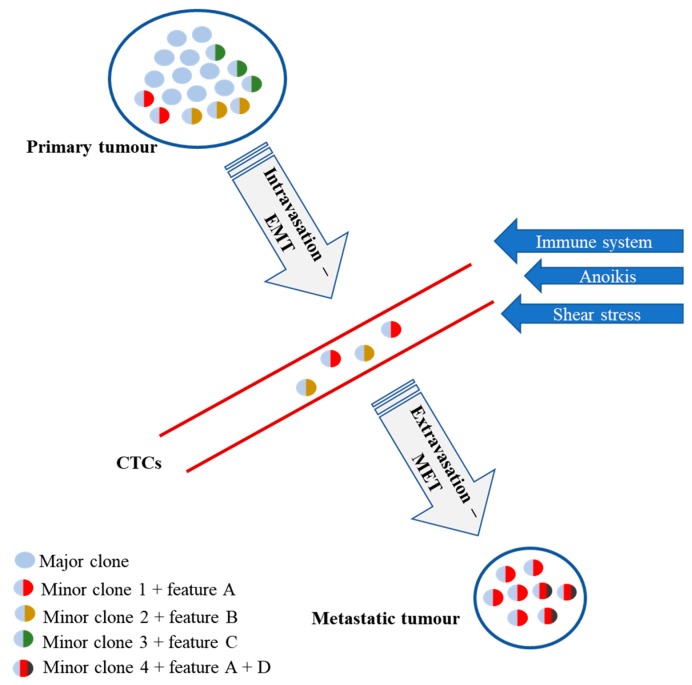
Heterogeneity of circulating tumour cells (CTCs). Major clone and minor clone 3 of the primary tumour do not shed CTCs, thus do not contribute to metastases. Two minor clones (1 and 2) with acquired genotypic and phenotypic changes A and B disseminate via hematogenous/lymphatic pathway, facing several stress insults. Only minor clone 1 is able to quit the blood/lymph stream and form metastasis in a distant site, where it acquires additional changes (D), generating minor clone 4. EMT, epithelial-mesenchymal transition; MET, mesenchymal-epithelial transition.

The main cause of invasive breast carcinoma (IBC)-related deaths is systemic spread of cancer cells and formation of metastases. These arise from minor subpopulations characterized by specific phenotypic traits, not representative of the bulk of primary tumour, and thus likely no to be targeted by the administered therapy (Figure 1) [5,6,7]. Tumour heterogeneity might, therefore, cause misinterpretation of therapeutic effects and should be appropriately addressed in evaluation of response to the treatment [8,9]. The need for monitoring of disease course and real-time assessment of therapy efficacy led to development of the so-called ‘liquid biopsies’, which would allow for early identification of disease progression (prognostic value) and selection of therapy (predictive value) [10,11,12,13]. Analysis of circulating tumour DNA (ctDNA), free circulating microRNAs (fc-miRNAs) and circulating tumour cells (CTCs), as major types of ‘liquid biopsy’, has generated interest in such personalized therapeutic approach [10,11,12,14,15,16,17]. Due to relatively small fragment size/amount of DNA, ctDNA can be used only for defining genomic changes related to cancer, such as mutation status or copy number variations, but not for detection of chromosomal aberrations [10,11,13,18,19]. Development of fc-microRNAs panels of prognostic or predictive value is the highlight of current translational research, however it provides only scarce information about tumour heterogeneity [20,21,22,23]. This can be assessed uniquely in the examination of CTCs. In IBC, the majority of therapeutic decisions is based on transcriptomic (gene expression), proteomic (status of HER2 and hormone receptors), or genomic profiling of cancer cells [24,25,26]. The aim of this review is to summarize current state of knowledge regarding multifactorial proteomic, genomic and transcriptomic panels applied to the analysis of CTCs and, when available, their potential clinical utility in IBC.

## 2. Multifactorial Prognostic and Predictive Panels in Invasive Breast Cancer

Assessment of ER, PR, HER2, and Ki67 statuses allows to classify IBC into four biologically and clinically distinct subtypes: luminal A, luminal B, HER2-enriched, or triple negative [27,28,29]. This so-called intrinsic IBC classification has been reproduced by hierarchical cluster analyses of gene expression profiles, favoured by modern approach to IBC subtyping, enabling a more comprehensive evaluation of mRNA levels of genes included in molecular signatures established in microarray experiments [27,30,31,32]. The PAM50 is the most widely used IBC gene signature and is considered a robust counterpart of classification based on immunohistochemical subtyping [30,33,34,35,36,37]. In the last years, many more predictive panels have been constructed and introduced to the management of IBC patients [32,38,39,40,41]. They were developed on the basis of either PAM50 genes (e.g. Prosigna) or genes selection guided by the outcome data (e.g., 70-gene signature—Mammaprint, 21-gene recurrence score—OncotypeDx, Breast Cancer Index, and multigenetic Endopredict) [38,39,40,41,42,43,44,45]. The actual advantage of multigenetic expression panels over routinely used predictive factors is still being disputed; however, according to both the World Health Organization’s (WHO) and St. Gallen’s expert consensus, multigenic panels are regarded as, at least, equally valuable (Table 1) [2,44].

## 3. Inter- and Intratumoural Heterogeneity of IBC

IBC is a highly heterogeneous disease and this heterogeneity is apparent between and within tumour-bearing patients (the “intertumoural” and “intratumoural” heterogeneity, respectively) [27,28,29,31,37,46]. The intertumoural heterogeneity of IBC concerns various histopathological classes, tumour grades or the aforementioned intrinsic molecular subtypes. As in all cancers, the intratumoural heterogeneity of IBC results from the dynamic changes that occur during tumour development, which involve gradual accumulation of diverse genetic, epigenetic, and phenotypic transformations combined with clonal expansion and selection (Figure 1) [46,47,48,49]. Progression into metastatic disease is associated with further phenotypic alterations, which are transient and reversible, so the similarity between primary tumours and synchronous metastases naïve to treatment can be expected and, indeed, is often observed [50,51]. The acquired transient molecular traits attributable to the process of dissemination remain poorly characterized, leaving a question mark against phenotypic concordance of circulating cells ‘in transit’ with primary or secondary tumours, or both [46,47,48,49,52,53]. The analysis of CTCs, so-called ‘liquid biopsy’ would, therefore, give an insight into the biology of the metastatic cascade providing ‘the missing link’ in molecular characterisation of tumour evolution. Moreover, it may serve as a valid counterpart for the assessment of prognostic and predictive factors in patients with IBC. This would be of prime importance for clinical practice, especially in advanced disease, where analysis of metastatic cells should provide information essential for identification of therapeutic targets, otherwise unobtainable from primary tumours.

## 4. CTCs—Clinical Potential and Limitations

The existence of CTCs was first proposed in the nineteenth century [54], but due to technological obstacles, the experimental examination of CTCs has become feasible only in recent years [52,55]. Although representative of only a small population of the primary tumour, CTCs are regarded as the prime means of disease dissemination and understanding of their biology is essential. Phenotypically CTCs are highly heterogeneous. This is due to the ways of entering the bloodstream (active vs. passive) but, above all, to molecular traits transiently acquired to meet demands of the rapidly changing microenvironment. Furthermore, CTCs subpopulation contains single cells as well as clusters [56,57,58], which exhibit intermediate EMT-phenotype, i.e., pro-survival and migratory abilities of mesenchymal cells and cell-cell interaction profile of epithelial cells [12]. As demonstrated by Aceto et al., these features might be responsible for up to 50-times higher metastatic abilities of CTC clusters than single cells [57]. Only 0.1% of single CTCs survive more than 24 h in the bloodstream (the half time of CTCs is estimated to range from 1 to 3 h), and only less than 0.01% of these cells have the ability to produce metastasis [48,49,59,60]. Identification of this most aggressive, metastasis initiating population of CTCs, represents both challenge and promise of modern diagnostics.

## 5. Methods of CTCs isolation

A number of methods have been developed for CTCs enrichment and isolations, with comprehensive summary published in a recent review [61]. CTCs analysis can be performed on enriched (with depletion of blood cells) or non-enriched samples (without depletion of blood cells), depending on the CTCs detection strategy, the throughput of the method and the type of marker to be analysed (DNA, RNA, or protein). Because gene expression profiles are usually tested in the bulk samples, high CTCs enrichment is preferred, what reduces background signals from the contaminating blood cells [62,63,64]. When proteins are analysed, each cell positive for the selected CTCs markers (potential CTC) is visually inspected, so the enrichment allows for reducing the number of cells to be screened and application of lower-throughput methods. 

Enrichment of IBC CTCs can be achieved by either positive or negative selection. In the positive selection (using e.g., CellSearch, AdnaTest, MACS, MagSweeper technologies), epithelial surface markers such as EpCAM are used (additionally MUC-1 in the AdnaTest) [65,66,67,68]. Elimination of mesenchymal-like CTCs, cells that lost epithelial features, i.e. undergone complete or partial EMT, from the sample is a severe limitation of the method [69]. In the negative selection, blood cells are removed by depletion of cells positive for CD45 [62,70] or a panel of markers: CD45, CD16, CD19, CD163, CD235a/GYP (as in MINDEC strategy) [71]. Although the purity of the sample may get compromised (the main limitation of the method), enrichment of cancer cells independently of their EMT status is the method’s great advantage. Isolation of mesenchymal CTCs is also possible in non-enriched CTCs samples (whole blood or PBMC blood fraction), where the risk of CTCs loss in a process of positive or negative selection is limited. However, in practice, these methods rely mostly on epithelial markers (most often epithelial cytokeratins) for detection of CTCs (e.g., CytoTrack [72], FASTCell™ [73], HD-CTC [74]). Inclusion of antibodies against specific mesenchymal markers in the CTCs staining panel would largely improve performance of these methods.

For genomic and transcriptomic characterization of CTCs, the purity of the CTCs-enriched fraction is the most important factor, since background signal might interfere with the readout of the molecular assay. For genomic test, the sensitivity of the assay for mutation detection in the background of wild-type allele should be considered [75,76]; though, in practice, genomic testing of CTCs is usually performed at the single cell level. In the transcriptomic assays, expression of genes included in the molecular test should be analysed in a number of control samples to adjust for background expression level and apply a cut-off value, when appropriate [62,63,64,77].

## 6. The Prognostic and Predictive Value of CTCs Detection and Quantification in Patients with IBC

The most aggressive clone in the primary tumour does not necessarily arise from the most abundant population of tumour cells, thus it is vital to reveal the ‘identity’ of clones with a high metastatic potential (Figure 1) [78,79]. As CTCs are being identified with these highly invasive and usually infrequent yet responsible for spread of disease cells, clinical trials are being designed to detect and assess CTCs for clinical decision making. The majority of studies of circulating cells have been focused on the possible prognostic and predictive value of CTCs detection and quantification. CTCs presence has been found an independent poor prognostic factor IBC [52]. Bidard et al. conducted an individual data analysis of 1944 metastatic IBC patients and showed an independent prognostic association between CTCs quantity and both progression-free (PFS) and overall survival (OS) [14]. This was confirmed in a large systematic review with meta-analysis by Yan et al., who included 6712 IBC patients from 50 studies and showed a strong predictive value of CTCs status during the treatment for both PFS and OS [80]. The count of CTCs correlated negatively with treatment results in IBC patients and their presence either before or after chemotherapy was linked with a poor prognosis [52,80,81,82,83,84,85,86,87,88,89]. 

Despite very promising results on prognostic value of CTCs quantification and detection, modification of applied therapy based on CTCs’ enumeration was found of no impact on OS and even PFS of patients in the, so far, most important SWOG0500 trial [90]. However, there were some doubts regarding the design of the study, as there were strong discrepancies in chemotherapeutic regimens applied in patients from different study arms, which puts in question the actual predictive value of CTCs quantification. In summary, the available data strongly suggest that not only a quantitative evaluation, but also a detailed characterization of CTCs’ biological traits is required to justify their future clinical application as close counterparts of primary or metastatic tumours, or both.

## 7. Assessment of Multiparametric Panels in IBC CTCs

### 7.1. Protein Level

On the protein level, practical assessment of CTCs’ biological features is currently limited to the key phenotypic IBC characteristics features (expression of ER, PR, and HER2) [73,91,92,93,94,95]. Majority of the studies reports a considerable disparity in the ER/PR/HER2 status between CTCs and primary or metastatic tumour cells but accumulated data are conflicting [53,91,92,93,96,97,98,99,100,101]. Aktas et al. and Babayan et al. demonstrated that expression of ER and PR in CTCs was lost or downregulated when compared to the ER/PR positive primary or metastatic tumours [92,93]. This finding was not confirmed by Kalinsky et al. and Fehm et al., who reported a similarity of the ER/PR profile between CTCs and primary/metastatic tumours [91,102]. Complicating matter even further, a recent analysis by Jordan et al. demonstrated that subsets of cells displaying opposite phenotypic features, e.g., HER2-positive and HER2-negative, may exist simultaneously among the CTCs population from the same patient [53]. 

Assessment of heterogeneity of CTCs is currently considered the biggest challenge in evaluation of a true predictive value of IBC CTCs for clinical practice [49,103,104]. The research aimed at valuation of the prognostic or predictive usefulness of CTCs’ ER/PR/HER2 status is limited. The biggest and most up-to-date study by Beije et al. [105,106,107,108,109] did not show either predictive or prognostic role of ER or HER2 status in 154 patients with metastatic IBC and this was confirmed by other reports [93,101,103,105]. In contrast, studies by Onstenk et al., Wallwiener et al., Hayashi et al., Paoletti et al. (inventor of a CTC-Endocrine therapy index (CTC-ETI)) or a meta-analysis by Wang et al. showed HER2 or ER levels in IBC CTCs as weak, but significant prognostic and predictive indicators [106,107,108,109,110]. To summarize, although technologically feasible, evaluation of ER/PR/HER2 status in IBC CTCs has not proved yet to be of a reliable prognostic and predictive value (Table 1) [93,104]. 

### 7.2. DNA Level

Analysis of mutations or copy number alterations in CTCs’ genomes can inform about oncogene addiction, sensitivity to treatment or mechanism of resistance to therapy. Thus, monitoring of changes in CTCs genotype during therapy administration might provide an invaluable insight into early signs of therapy failure. Since genomic testing is performed most often in single CTCs, reliable protocols and tools need to be applied to minimize the possibility of false positive results due to single-cells DNA amplification errors (reviewed in [141]). 

Currently there are no pre-defined multiparametric panels for evaluation of genomic changes in single cells. Single cells analysis is mainly performed after CellSearch enrichment, when cells are flushed out of the cassette and loaded onto DEPArray for single cell recovery. The majority of the analysis is based on Whole Genome Amplification (WGA) of single cells and further Sanger sequencing of selected hot spot regions. In all the studies described below, WGA was performed using Ampli1 WGA assay (Silicon Biosystems), which relies on enzymatic genome digestion with *Mse I*, adapter ligation and further PCR-based fragments amplification. A number of studies testing mutations in CTCs are restricted to the analysis of a single gene in CTCs (e.g., *TP53*, *PIK3CA*, *HER2*, *ESR1*) [13,142,143,144,145,146]. Polzer et al. tested *HER2* amplification (by qPCR) and *PIK3CA* mutations (by Sanger sequencing) in exon 9 and 20 in CTCs isolated from breast cancer patients (stage I-IV) with CellSearch-enrichment and DEPArray single cells recovery. They have found that *HER2* was amplified in CTCs of 16.7% of the patients, with 16 cases (out of 17) showing homogenous *HER2* amplification status, indicative of CTCs clonality [112]. Disparity in the *HER2* status between CTCs and matched primary tumours reached 20%. Mutations in *PIK3CA* gene were found in 37.2% of the patients and showed heterogeneous CTCs mutation status in 9 out of 17 patients [112]. Disparity in the *PIK3CA* mutation status between CTCs and matched primary tumours was higher than that of *HER2* and concerned 66% of the cases. Prior to administration of HER2-targeted therapies, in 28.6% of the patients, *HER2*-amplified CTCs had *PIK3CA* mutations, known to be associated with resistance to anti-HER2 treatment and worse outcome of patients with HER2-positive tumours [111,147,148]. Lower rate of *PIK3CA* mutation in exons 1, 9, and 20 was observed in the study by Neves et al., who have tested MoFlo XDP cell sorter-isolated single CTCs obtained after CellSearch-enrichment of blood samples from metastatic breast cancer patients [117]. Mutated *PIK3CA* (tested by Sanger sequencing) was detected in 16% of the patients and no mutations in *TP53* (exon 5, 7, 8) were found. Copy number alterations, such as frequent amplification of *CCND1* locus (46% of CTCs), tested by aCGH and further validated by qPCR, were characteristic of metastatic breast cancers. 

De Luca et al. used targeted sequencing (on IonTorrent PGM system) of a pre-defined set of 50 oncogenes and tumour suppressor genes with a use of Ion AmpliSeq™ Cancer Hotspot Panel v2 in CellSearch-enriched and DEPArray isolated single CTCs from four metastatic breast cancer patients [120]. Overall, 51 sequence variants were found in 25 genes. Observed mutations were mostly not shared among CTCs from the same patient (3–5 CTCs were analysed per patient), apart from mutations in *HER2, TP53, KIT*, and *PDGFRA* genes, which occurred in at least two CTCs from the same patient. Some of the recorded mutations, such as *TP53* p.R273C, have previously been connected with more aggressive phenotype and found in vitro to be associated with increased cell proliferative and migratory potential as well as drug resistance [149]. Mutation in *HER2* (ErbB2-p.V777L) was described to confer decreased sensitivity to lapatinib in vitro [150]. Sequencing of three available matched primary tumours (for mutations found in CTCs) revealed shared changes in *PDGFRA* (benign change) in one patient and *TP53* mutations in two other patients. The rest of the variants were not present in the primary tumours. In one case, analysis of CTCs performed before and one month after treatment initiation revealed existence of CTCs with on-treatment acquired mutations in *EGRF, PIK3CA, SMAD4, SMARCB1,* and *VHL* genes. However, detected mutations were present only in one out of three CTCs, indicating lack of clonality. Targeted sequencing (on IonTorrent PGM system) with Ion AmpliSeq™ Cancer Hotspot Panel v2 was used also by Shaw et al. in five metastatic breast cancer patients with high CellSearch CTCs count (>100) [10]. Single CTCs were isolated with the DEPArray system. Mutations (in at least two CTCs from the same patient) were observed in four genes, *PIK3CA, TP53, ESR1*, and *KRAS,* with the last two present in CTCs, but not in the matched primary tumours. Since *KRAS* mutation is related to worse prognosis of breast cancer patients, its presence in CTCs can potentially be used in therapy selection [151]. Interestingly, serial sampling of blood in one patient and concomitant analysis of ctDNA showed that presence of *KRAS* mutation (p.G12D), not detected in the primary tumour, was found in both CTCs and ctDNA, whereas low frequency mutation in *TP53* (p.P278R) was detected only in ctDNA. As suggested by the authors, discordance between ctDNA and CTCs might reflect the existence of distinct metastatic clones giving rise to either CTCs or ctDNA. If so, identification of the origin of metastasis initiating population (sharing mutations with ctDNA or CTCs) and parallel analysis of CTCs and ctDNA would have significant clinical implications. In another study by Bingham et al., CellSearch-enriched, DEPArray isolated single CTCs or CTCs clusters from metastatic breast cancer were analysed for mutations in *TP53, RB1, PIK3CA*, and *HER2* using Sanger sequencing or NGS (only mutation in *RB1* gene) [118]. In every case, mutations found in clusters of CTCs were also observed in at least one single CTCs from the same patient. Mutations were chosen based on their occurrence in the matched primary tumours or metastases, thus confirming (or not) the existence of primary/metastatic tumour- derived clones in CTCs. In one patient three genes were mutated in CTCs: *PIK3CA* K111E, *HER2* S310F and V777L, as well as *TP53* C229 fs*10, in another case two mutations were observed: *TP53* R110 delC fs*13 and *RB1* K720*. For other three women, only one mutation was found in CTCs—either *RB1* 607+1 G > C, *TP53* R110 delG fs*13, or *TP53* P190_H193 > *E. In five out of six cases, mutation status results were concordant between CTCs and matched primary/metastatic tumour, however CTCs often showed heterogeneous mutation pattern. In one case, *TP53* mutation found in the chest wall metastases was not detected in five tested CTCs and one CTCs cluster. Since mutations in *TP53* were correlated with the poorer response to anthracycline- or tamoxifen-based treatment as well as sensitivity to paclitaxel, detection of CTCs carrying *TP53* mutations might provide important predictive information [152,153,154,155]. Hetero- and homozygous *TP53* mutation R248W (linked with aggressive characteristics and drug resistance) in single CTCs and CTCs clusters from one ER+/PR-/HER2- metastatic breast cancer patient, confirmed in the matched primary tumour, was also reported by Mu et al. [119]. No activating mutations in *ESR1* (exon 8 (c.1607-1621)) were observed in either single CTCs, CTCs clusters (isolated by DEPArray after ScreenCell® Cyto device enrichment) or primary tumour of that patient.

Overall, genomic testing of CTCs holds a great promise as a valid tool to monitor disease progression in breast cancer patients (Table 1). However, assessment of its true clinical utility relies on the follow up data and meaningful statistical analyses, lacking from currently available reports.

### 7.3. RNA Level

Current technology of CTCs enrichment allows for more reliable and easier measurement of RNA than proteins [60,156]. In IBC, information provided by numerous reports on CTCs is limited to single or a small number of genes, but technology for assessment of multiple genetic panels is becoming widely available [73,96,104,126,129,156]. 

So far, most of the studies have evaluated mRNA expression of key IBC predictive biological genes (*ESR1* (ER), *PGR* (PR), *ERBB2* (HER2)) in CTCs [99,102,107,121,122,157,158,159]. The major findings were consistent with those for ER/PR/HER2 proteins, with most studies highlighting an intratumoural heterogeneity between IBC CTCs and primary or metastatic tumours [99,102,157,158,159]. Predictive and prognostic values of *ESR1* or *ERBB2* mRNA levels in CTCs were shown to have closer association with PFS and OS than their protein counterparts [107,109,121,122].

The other group of studies on mRNA in CTCs focused on biological aspect of CTCs, in particular those related to tumour evolution. These dynamic changes encompass several fundamental molecular and phenotypic adaptations, including EMT, acquisition of stem-like features, followed by the EMT reversal, the mesenchymal-to-epithelial transition (MET), required for the formation of a secondary tumour at the ‘destination site’ [160,161,162]. Several reports showed that expression of EMT markers and stem-like traits were up-regulated in IBC CTCs [62,97,98,99,124,163,164]. It must be noted that, similarly to ER/PR/HER2, heterogeneity among IBC CTCs in both stemness and EMT markers levels is also extremely high [12,17,165,166]. Armstrong et al. showed that even 75% of IBC CTCs in metastatic patients simultaneously co-express mesenchymal and epithelial markers. Yu et al. demonstrated dynamic changes in the levels of EMT markers in serial examination and Guan et al. found that expression of ER and PR varied between subpopulations of CTCs expressing different levels of EMT markers [12,159,167].

Because CTCs represent a small but a composite and heterogeneous population of cancer cells, their molecular characterization using small groups of routine markers may not be adequate or sufficient [60,168]. Complex panels that would depict biology of cancer and have a documented clinical value seem essential for the optimal evaluation of CTCs’ metastatic abilities [104]. Several studies of molecular gene signatures in IBC CTCs presented encouraging results and showed that analysis of some multigenetic profiles is robustly possible [98,128,129,130,156,169,170]. However, provided information is insufficient from both the biological and clinical point of view, i.e. the studies analysed small number of patients, did not include all genes from the multigene profiles of known predictive/prognostic value (e.g., PAM50, Nanostring, Mammaprint, OncotypeDx, Endopredict), were limited to technical or innovative aspects of multiparametric panels [60,98,126,128,129,130,156,168,169,170,171,172,173]. In addition, small amounts of CTCs RNA and contamination of enriched samples with residual leukocyte RNA often results in non-conclusive results of multiparametric tests, designed originally for larger tissue samples (primary tumour). For example, PAM50 or Prosigna panels could not have been successfully examined in IBC CTCs in studies by Lang et al. and Porras et al. [126,127]. Summarizing, the reports on examination of the clinically and biologically valid gene signatures in CTCs from IBC patients are scarce, e.g., no successful comprehensive analysis of intrinsic molecular subtypes of CTCs in relation to both primary and metastatic foci using a biologically and clinically relevant gene signature has been reported yet (Table 1) [60,126,129,130]. 

### 7.4. microRNA Level

Free circulating microRNA, a stable, easily isolated, and measurable subtype of RNA, has a unique clinical potential and is the highlight of current translational ‘liquid biopsies’ research [21,22,23]. In IBC, plasma microRNA panels of potential biologic, predictive or prognostic value have been developed by a number of research groups [20,21,22,23]. In contrast, reports on microRNA profiling in isolated CTCs are far less common. Leong et al. and Gasch et al. described an optimized methodology for microRNA expression measurement in CTCs either by subtracting of plasma microRNA background or by application of in situ hybridization and the CellSearch CTC detection system [131,132]. Sieuwerts et al. demonstrated a panel of 10 microRNAs that were upregulated particularly in IBC CTCs [128]. These few publications show that the measurement of microRNAs level in IBC CTCs is uncommon, mainly due to technological difficulties. These may be overcome by emerging novel single cell-based approaches that should provide a powerful tool for accurate analysis of microRNA profiles in enriched CTCs [133].

## 8. Single Cell Analysis of CTCs—Future or Confusion?

Development of technologies for single cell isolation and genome/transcriptome amplification enabled molecular profiling of single CTCs [133,174,175]. This approach to CTCs analysis, allowing assessment of disease heterogeneity in the limited sample material, is recently gaining wide recognition [120,133,143]. Since molecular characteristics of CTCs are bound to be related to the response to therapy, evaluation of individual CTCs might provide valuable information for tailored treatment. CTCs are known to have different metastatic abilities, which implies that not only the quantity, but also the qualitative features of CTCs are important for estimation of the risk of disease progression [12,16,17,62,176]. Therefore, combination of genomic and transcriptomic profiling techniques from a single cell, such as DRseq [177], G&T-seq [178], as well as proteomic and transcriptomic [179] single cell interrogation open new avenues for clinically oriented research. Recent reports suggest a direct possibility of application of single cell technologies for personalized treatment guidance for IBC patients [133,180].

The main disadvantages of single cells techniques include the cost, data redundancy, and technical limitations interfering with interpretation of the results. A crucial step in single cell analysis is elimination of low-quality cells/material, as they might bias the results [181]. Loss of material during single cell sample preparation or due to genetic material degradation (especially RNA) at the time of sample collection might give false negative results of gene expression or mutation status. Thus, it is of utmost importance to distinguish inherent heterogeneity of CTCs mutational status from technical errors, such as detection of mutations introduced during sample preparation or allelic drop-out. Moreover, stochastic nature of the transcription process (so called transcriptional burst), together with the dynamic changes in cellular mRNA levels due to degradation, might lead to high cell-to-cell variation, not observed in the analysis of cell pools [182,183]. Therefore, analysis of multiple redundant markers would increase the reliability of the results as would sequencing of multiple cells from the same patients, but this would have to be balanced against heterogeneity of the cells. In NGS technologies, sequencing depth has also to be considered; in single cell RNA profiling it has been estimated that one million reads per cells generates satisfying coverage rates to detect low-level transcripts expression [184]. 

## 9. Conclusions

CTCs in peripheral blood, the so called ‘liquid biopsy’, are being considered nowadays as a valid counterpart of prognostic and predictive factors in IBC. Molecular characterisation of CTCs is of particular importance in advanced disease, where analysis of metastatic cells should provide information essential for identification of therapeutic targets, otherwise unobtainable from primary tumours. If CTCs are truly representative of metastases in terms of known clinico-pathological factors, characterization of the molecular profile of this easily accessible ‘biopsy’ might be of prime importance for clinical practice. Since CTCs show substantial heterogeneity in clinically relevant markers, single cells analysis seems to be a mandatory approach for accurate characterisation of systemically spread cancer and assessment of clinical utility and validity of CTCs-guided therapies. Although, from the technological point of view, we may be ready for the ‘liquid’ revolution, translational and clinical research, mainly well designed randomized clinical trials, is urgently needed to allow evaluation of the true value of the detailed CTCs examination in relation to known prognostic and predictive disease features. 

## Figures and Tables

**Table 1:** Prognostic and predictive value of biological markers in invasive breast carcinoma—primary tumour vs. CTCs. IBC, invasive breast carcinoma; CTCs, circulating tumour cells; PFS, progression-free survival; OS, overall survival.

Analyte	Marker	Prognostic/Predictive Value in IBC-Primary Tumour	Successfully Applied in CTCs	Prognostic or Predictive Value when Examined in CTCs	Reference
DNA	*HER2* amplification	Strong predictive value.	Yes, can be robustly assessed.	No confirmed prognostic/predictive value in metastatic breast cancer patients treated with ado-trastuzumab emtansine [111].	[112,113,114,115,116]
	*PIK3CA* gain-of-function mutation	Prognostic factor linked to good prognosis; not applied in routine clinical practice.	Yes, can be robustly assessed.	Not assessed.	[112,117,118]
	*TP53* loss-of-function mutation	Prognostic factor linked to poor prognosis; no predictive value in routine clinical practice.	Yes, can be robustly assessed.	Not assessed.	[117,119]
	*RB1*	Prognostic factor linked to poor prognosis. Predictive value—low *RB*1 expression in triple negative/ER-negative breast cancers related to good prognosis in patients treated with chemotherapy.	Yes, can be assessed.	Not assessed.	[118]
	*ESR1* mutations	Prognostic factor linked to poor prognosis, potentially to be applied in clinics as a negative predictive factor (hormone resistance).	Yes, can be robustly assessed.	Not assessed.	[119]
	Ion AmpliSeq™ Cancer Hotspot Panel v2	Not assessed.	Yes, can be robustly assessed.	Not assessed.	[10,120]
RNA	ESR1/PGR	Both receptors routinely examined at protein level. Discrepancies between mRNA and protein expression frequently observed, but mRNA evaluation also shown of prognostic/ predictive value.	Yes, can be robustly assessed.	Prognostic value like in primary tumour, discrepant results of predictive value.	[93,105,107].
	HER2	Discrepancies between mRNA and protein levels seen in nearly 25% of patients. Protein examination routinely applied in clinics. mRNA also of both prognostic and predictive value.	Yes, can be robustly assessed.	HER2-positive CTCs are linked to poor prognosis in terms of both OS and PFS.	[108,109,121,122,123]
	EMT pathway molecules	Association between high levels of mesenchymal markers frequently reported. No predictive value or validated clinical application.	Yes, but efficiency of protocol/s still to be improved.	High frequency of mesenchymal CTCs linked to poor prognosis. No data on predictive value.	[62,97,99,124,125]
	PAM50	Prognostic and predictive value comparable to standard predictive factors, useful in clinical practice.	No report on coverage of all genes; single reports on partial assessment of the signature	Not assessed.	[91,126,127,128]
	Prosigna	Routinely applied predictive panel in clinics.	No, cannot be robustly applied.	Not assessed.	[126,127]
	Other panels, including EndoPredict, Mammaprint, OncotypeDx, Breast Cancer Index	Each panel designed to predict outcome; prognostic and predictive values of various panels similarly high across several comparing studies; routinely applied in clinics.	No reports so far.	Not assessed.	[60,126,129,130]
	microRNAs	Some panels of prognostic value when measured in primary tumour, but the known panels mostly applied for free-circulating microRNAs.	On-going research to resolve technical issues.	Not assessed.	[128,131,132,133]
Protein	ER, PR	The most significant prognostic and predictive factors applied in clinics.	Yes, can be robustly assessed.	Prognostic value.	[11,101,103,105,106,107,108]
	HER2	One of the key prognostic and predictive factors applied in clinics.	Yes, can be robustly assessed.	Poor prognostic value in terms of PFS in patients with HER2-positive CTCs in comparison to patients with HER2-negative CTCs, no strong prognostic value regarding OS.	[101,105,106,109]
	Ki67	One of the key prognostic and predictive factors applied in clinics.	Yes, but some technical difficulties still to be overcome.	Not assessed.	[134,135]
	EMT pathway molecules	Prognostic role of E-cadherin, vimentin and keratins.	Yes, can be robustly assessed	EMT activation related with reduced PFS and OS in metastatic patients.	[16,136,137,138]
	Proteomic panels	Prognostic significance of breast cancer subtypes identified by a multi-protein marker set.	Yes, can be assessed.	Not assessed. Used in basic science research.	[139,140]

## References

[1] Ferlay J., Soerjomataram I I., Dikshit R., Eser S., Mathers C., Rebelo M., Parkin D.M., Forman D D., Bray F. (2014). Cancer incidence and mortality worldwide: Sources, methods and major patterns in GLOBOCAN 2012. Int. J. Cancer.

[2] Sinn H.-P., Kreipe H. (2013). A brief overview of the WHO classification of breast tumors, 4th edition, focusing on issues and updates from the 3rd edition. Breast care.

[3] Ferlay J., Colombet M., Soerjomataram I., Mathers C., Parkin D.M., Piñeros M., Znaor A., Bray F. (2018). Estimating the global cancer incidence and mortality in 2018: GLOBOCAN sources and methods. Int. J. Cancer.

[4] Bray F., Ferlay J., Soerjomataram I., Siegel R.L., Torre L.A., Jemal A. (2018). Global cancer statistics 2018: GLOBOCAN estimates of incidence and mortality worldwide for 36 cancers in 185 countries. CA. Cancer J. Clin..

[5] Allegra J.C., Barlock A., Huff K.K., Lippman M.E. (1980). Changes in multiple or sequential estrogen receptor determinations in breast cancer. Cancer.

[6] Lindström L.S., Karlsson E., Wilking U.M., Johansson U., Hartman J., Lidbrink E.K., Hatschek T., Skoog L., Bergh J. (2012). Clinically used breast cancer markers such as estrogen receptor, progesterone receptor, and human epidermal growth factor receptor 2 are unstable throughout tumor progression. J. Clin. Oncol..

[7] Wu J.M., Fackler M.J., Halushka M.K., Molavi D.W., Taylor M.E., Teo W.W., Griffin C., Fetting J., Davidson N.E., De Marzo A.M. (2008). Heterogeneity of breast cancer metastases: comparison of therapeutic target expression and promoter methylation between primary tumors and their multifocal metastases. Clin. Cancer Res..

[8] Polyak K. (2011). Heterogeneity in breast cancer. J. Clin. Invest..

[9] Turashvili G., Brogi E. (2017). Tumor heterogeneity in breast cancer. Front. Med..

[10] Shaw J.A., Guttery D.S., Hills A., Fernandez-Garcia D., Page K., Rosales B.M., Goddard K.S., Hastings R.K., Luo J., Ogle O. (2017). Personalized medicine and imaging mutation analysis of cell-free DNA and single circulating tumor cells in metastatic breast cancer patients with high circulating tumor cell counts. Clin. Cancer Res..

[11] Rossi G., Mu Z., Rademaker A.W., Austin L.K., Strickland K.S., Costa R.L.B., Nagy R.J., Zagonel V., Taxter T.J., Behdad A. (2018). Cell-free DNA and circulating tumor cells: comprehensive liquid biopsy analysis in advanced breast cancer. Clin. Cancer Res..

[12] Yu M., Bardia A., Wittner B.S., Stott S.L., Smas M.E., Ting D.T., Isakoff S.J., Ciciliano J.C., Wells M.N., Shah A.M. (2013). Circulating breast tumor cells exhibit dynamic changes in epithelial and mesenchymal composition. Science (80-).

[13] Guttery D.S., Page K., Hills A., Woodley L., Marchese S.D., Rghebi B., Hastings R.K., Luo J., Pringle J.H., Stebbing J. (2015). Noninvasive detection of activating estrogen receptor 1 (ESR1) mutations in estrogen receptor-positive metastatic breast cancer. Clin. Chem..

[14] Bidard F.-C., Peeters D.J., Fehm T., Nolé F., Gisbert-Criado R., Mavroudis D., Grisanti S., Generali D., Garcia-Saenz J.A., Stebbing J. (2014). Clinical validity of circulating tumour cells in patients with metastatic breast cancer: A pooled analysis of individual patient data. Lancet. Oncol..

[15] Dawson S.-J., Tsui D.W.Y., Murtaza M., Biggs H., Rueda O.M., Chin S.-F., Dunning M.J., Gale D., Forshew T., Mahler-Araujo B. (2013). Analysis of circulating tumor DNA to monitor metastatic breast cancer. N. Engl. J. Med..

[16] Papadaki M.A., Stoupis G., Theodoropoulos P.A., Mavroudis D., Georgoulias V., Agelaki S. (2018). Circulating tumor cells with stemness and epithelial-to-mesenchymal transition features are chemoresistant and predictive of poor outcome in metastatic breast cancer. Mol. Cancer Ther..

[17] Markiewicz A., Nagel A., Szade J., Majewska H., Skokowski J., Seroczynska B., Stokowy T., Welnicka-Jaskiewicz M., Zaczek A.J. (2018). Aggressive phenotype of cells disseminated via hematogenous and lymphatic route in breast cancer patients. Transl. Oncol..

[18] Shaw J.A., Page K., Blighe K., Hava N., Guttery D., Ward B., Brown J., Ruangpratheep C., Stebbing J., Payne R. (2012). Genomic analysis of circulating cell-free DNA infers breast cancer dormancy. Genome Res..

[19] Neumann M.H.D., Bender S., Krahn T., Schlange T. (2018). ctDNA and CTCs in liquid biopsy - current status and where we need to progress. Comput. Struct. Biotechnol. J..

[20] Kolacinska A., Morawiec J., Fendler W., Malachowska B., Morawiec Z., Szemraj J., Pawlowska Z., Chowdhury D., Choi Y.E., Kubiak R. (2014). Association of microRNAs and pathologic response to preoperative chemotherapy in triple negative breast cancer: Preliminary report. Mol. Biol. Rep..

[21] Piasecka D., Braun M., Kordek R., Sadej R., Romanska H. (2018). MicroRNAs in regulation of triple-negative breast cancer progression. J. Cancer Res. Clin. Oncol..

[22] Lin S., Gregory R.I. (2015). MicroRNA biogenesis pathways in cancer. Nat. Rev. Cancer.

[23] Kurozumi S., Yamaguchi Y., Kurosumi M., Ohira M., Matsumoto H., Horiguchi J. (2017). Recent trends in microRNA research into breast cancer with particular focus on the associations between microRNAs and intrinsic subtypes. J. Hum. Genet..

[24] Senkus E., Kyriakides S., Ohno S., Penault-Llorca F., Poortmans P., Rutgers E., Zackrisson S. (2015). Primary breast cancer: ESMO Clinical Practice Guidelines for diagnosis, treatment and follow-up † incidence and epidemiology. Ann. Oncol..

[25] Godet I., Gilkes D.M. (2017). BRCA1 and BRCA2 mutations and treatment strategies for breast cancer. Integr. Cancer Sci. Ther..

[26] Griffith O.L., Spies N.C., Anurag M., Griffith M., Luo J., Tu D., Yeo B., Kunisaki J., Miller C.A., Krysiak K. (2018). The prognostic effects of somatic mutations in ER-positive breast cancer. Nat. Commun..

[27] Sørlie T., Perou C.M., Tibshirani R., Aas T., Geisler S., Johnsen H., Hastie T., Eisen M.B., Van De Rijn M., Jeffrey S.S. (2001). Gene expression patterns of breast carcinomas distinguish tumor subclasses with clinical implications. Proc. Natl. Acad. Sci. USA.

[28] Sørlie T., Tibshirani R., Parker J., Hastie T., Marron J.S., Nobel A., Deng S., Johnsen H., Pesich R., Geisler S. (2003). Repeated observation of breast tumor subtypes in independent gene expression data sets. Proc. Natl. Acad. Sci. USA.

[29] Perou C.M., Sørlie T., Eisen M.B., van de Rijn M., Jeffrey S.S., Rees C.A., Pollack J.R., Ross D.T., Johnsen H., Akslen L.A. (2000). Molecular portraits of human breast tumours. Nature.

[30] Parker J.S., Mullins M., Cheung M.C.U., Leung S., Voduc D., Vickery T., Davies S., Fauron C., He X., Hu Z. (2009). Supervised risk predictor of breast cancer based on intrinsic subtypes. J. Clin. Oncol..

[31] Prat A., Perou C.M. (2011). Deconstructing the molecular portraits of breast cancer. Mol. Oncol..

[32] Kittaneh M., Montero A.J., Glück S. (2013). Molecular profiling for breast cancer: A comprehensive review. Biomark. Cancer.

[33] Wallden B., Storhoff J., Nielsen T., Dowidar N., Schaper C., Ferree S., Liu S., Leung S., Geiss G., Snider J. (2015). Development and verification of the PAM50-based Prosigna breast cancer gene signature assay. BMC Med. Genomics.

[34] Dowsett M., Sestak I., Lopez-Knowles E., Sidhu K., Dunbier A.K., Cowens J.W., Ferree S., Storhoff J., Schaper C., Cuzick J. (2013). Comparison of PAM50 risk of recurrence score with oncotype DX and IHC4 for predicting risk of distant recurrence after endocrine therapy. J. Clin. Oncol..

[35] Prat A., Parker J.S., Fan C., Perou C.M. (2012). PAM50 assay and the three-gene model for identifying the major and clinically relevant molecular subtypes of breast cancer. Breast Cancer Res. Treat..

[36] Tobin N.P., Lundberg A., Lindström L.S., Harrell J.C., Foukakis T., Carlsson L., Einbeigi Z., Linderholm B.K., Loman N., Malmberg M. (2017). PAM50 provides prognostic information when applied to the lymph node metastases of advanced breast cancer patients. Clin Cancer Res..

[37] Dai X., Li T., Bai Z., Yang Y., Liu X., Zhan J., Shi B. (2015). Breast cancer intrinsic subtype classification, clinical use and future trends. Am J Cancer Res.

[38] van de Vijver M.J., He Y.D., van’t Veer L.J., Dai H., Hart A.A.M., Voskuil D.W., Schreiber G.J., Peterse J.L., Roberts C., Marton M.J. (2002). A gene-expression signature as a predictor of survival in breast cancer. N. Engl. J. Med..

[39] van’t Veer L.J., Dai H., van de Vijver M.J., He Y.D., Hart A.A.M., Mao M., Peterse H.L., van der Kooy K., Marton M.J., Witteveen A.T. (2002). Gene expression profiling predicts clinical outcome of breast cancer. Nature.

[40] Cardoso F., van’t Veer L.J., Bogaerts J., Slaets L., Viale G., Delaloge S., Pierga J.-Y., Brain E., Causeret S., DeLorenzi M. (2016). 70-Gene signature as an aid to treatment decisions in early-stage breast cancer. N. Engl. J. Med..

[41] Paik S., Shak S., Tang G., Kim C., Baker J., Cronin M., Baehner F.L., Walker M.G., Watson D., Park T. (2004). A multigene assay to predict recurrence of tamoxifen-treated, node-negative breast cancer. N. Engl. J. Med..

[42] Nielsen T.O., Parker J.S., Leung S., Voduc D., Ebbert M., Vickery T., Davies S.R., Snider J., Stijleman I.J., Reed J. (2010). A comparison of PAM50 intrinsic subtyping with immunohistochemistry and clinical prognostic factors in tamoxifen-treated estrogen receptor-positive breast cancer. Clin. Cancer Res..

[43] Jerevall P.-L., Ma X.-J., Li H., Salunga R., Kesty N.C., Erlander M.G., Sgroi D.C., Holmlund B., Skoog L., Fornander T. (2011). Prognostic utility of HOXB13:IL17BR and molecular grade index in early-stage breast cancer patients from the Stockholm trial. Br. J. Cancer.

[44] Morigi C. (2017). Highlights from the 15th St Gallen International Breast Cancer Conference 15-18 March, 2017, Vienna: Tailored treatments for patients with early breast cancer. ecancermedicalscience.

[45] Filipits M., Rudas M., Jakesz R., Dubsky P., Fitzal F., Singer C.F., Dietze O., Greil R., Jelen A., Sevelda P. (2011). A new molecular predictor of distant recurrence in er-positive, HER2-negative breast cancer adds independent information to conventional clinical risk factors. Clin. Cancer Res..

[46] Kimbung S., Loman N., Hedenfalk I. (2015). Clinical and molecular complexity of breast cancer metastases. Semin. Cancer Biol..

[47] Chaffer C.L., Weinberg R.A. (2011). A perspective on cancer cell metastasis. Science.

[48] Fidler I.J. (2003). The pathogenesis of cancer metastasis: The “seed and soil” hypothesis revisited. Nat. Rev. Cancer.

[49] Joosse S.A., Gorges T.M., Pantel K. (2015). Biology, detection, and clinical implications of circulating tumor cells. EMBO Mol. Med..

[50] Aurilio G., Disalvatore D., Pruneri G., Bagnardi V., Viale G., Curigliano G., Adamoli L., Munzone E., Sciandivasci A., De Vita F. (2014). A meta-analysis of oestrogen receptor, progesterone receptor and human epidermal growth factor receptor 2 discordance between primary breast cancer and metastases. Eur. J. Cancer.

[51] Krøigård A.B., Larsen M.J., Thomassen M., Kruse T.A. (2016). Molecular concordance between primary breast cancer and matched metastases. Breast J..

[52] Braun S., Naume B. (2005). Circulating and disseminated tumor cells. J. Clin. Oncol..

[53] Jordan N.V., Bardia A., Wittner B.S., Benes C., Ligorio M., Zheng Y., Yu M., Sundaresan T.K., Licausi J.A., Desai R. (2016). HER2 expression identifies dynamic functional states within circulating breast cancer cells. Nature.

[54] Ashworth T. (1869). A case of cancer in which cells similar to those in the rumours were seen in the blood after death. Aust. Med. J..

[55] Allard W.J., Matera J., Miller M.C., Repollet M., Connelly M.C., Rao C., Tibbe A.G.J., Uhr J.W., Terstappen L.W.M.M. (2004). Tumor cells circulate in the peripheral blood of all major carcinomas but not in healthy subjects or patients with nonmalignant diseases. Clin Cancer Res..

[56] Mu Z., Wang C., Ye Z., Austin L., Civan J., Hyslop T., Palazzo J.P., Jaslow R., Li B., Myers R.E. (2015). Prospective assessment of the prognostic value of circulating tumor cells and their clusters in patients with advanced-stage breast cancer. Breast Cancer Res. Treat..

[57] Aceto N., Bardia A., Miyamoto D.T., Donaldson M.C., Wittner B.S., Spencer J.A., Yu M., Pely A., Engstrom A., Zhu H. (2014). Circulating tumor cell clusters are oligoclonal precursors of breast cancer metastasis. Cell.

[58] Cho E.H., Wendel M., Luttgen M., Yoshioka C., Marrinucci D., Lazar D., Schram E., Nieva J., Bazhenova L., Morgan A. (2012). Characterization of circulating tumor cell aggregates identified in patients with epithelial tumors. Phys. Biol..

[59] Meng S., Tripathy D., Frenkel E.P., Shete S., Naftalis E.Z., Huth J.F., Beitsch P.D., Leitch M., Hoover S., Euhus D. (2004). Circulating tumor cells in patients with breast cancer dormancy. Clin. Cancer Res..

[60] Magbanua M.J.M., Park J.W. (2017). Isolation and Molecular Characterization of Circulating Tumor Cells.

[61] Ferreira M.M., Ramani V.C., Jeffrey S.S. (2016). Circulating tumor cell technologies. Mol. Oncol..

[62] Markiewicz A., Ksia̧zkiewicz M., Wełnicka-Jaśkiewicz M., Seroczyńska B., Skokowski J., Szade J., Zaczek A.J. (2014). Mesenchymal phenotype of CTC-enriched blood fraction and lymph node metastasis formation potential. PLoS ONE.

[63] Sieuwerts A.M., Kraan J., Bolt-de Vries J., van der Spoel P., Mostert B., Martens J.W.M., Gratama J.-W., Sleijfer S., Foekens J.A. (2009). Molecular characterization of circulating tumor cells in large quantities of contaminating leukocytes by a multiplex real-time PCR. Breast Cancer Res. Treat..

[64] Mego M., Cierna Z., Janega P., Karaba M., Minarik G., Benca J., Sedlácková T., Sieberova G., Gronesova P., Manasova D. (2015). Relationship between circulating tumor cells and epithelial to mesenchymal transition in early breast cancer. BMC Cancer.

[65] Cristofanilli M., Budd G.T., Ellis M.J., Stopeck A., Matera J., Miller M.C., Reuben J.M., Doyle G.V., Allard W.J., Terstappen L.W.M.M. (2004). Circulating tumor cells, disease progression, and survival in metastatic breast cancer. N. Engl. J. Med..

[66] Andreopoulou E., Yang L.-Y., Rangel K.M., Reuben J.M., Hsu L., Krishnamurthy S., Valero V., Fritsche H.A., Cristofanilli M. (2012). Comparison of assay methods for detection of circulating tumor cells in metastatic breast cancer: AdnaGen AdnaTest BreastCancer Select/Detect^TM^ versus Veridex CellSearch^TM^ system. Int. J. Cancer.

[67] Pluim D., Devriese L.A., Beijnen J.H., Schellens J.H.M. (2012). Validation of a multiparameter flow cytometry method for the determination of phosphorylated extracellular-signal-regulated kinase and DNA in circulating tumor cells. Cytom. Part A.

[68] Talasaz A.H., Powell A.A., Huber D.E., Berbee J.G., Roh K.-H., Yu W., Xiao W., Davis M.M., Pease R.F., Mindrinos M.N. (2009). Isolating highly enriched populations of circulating epithelial cells and other rare cells from blood using a magnetic sweeper device. Proc. Natl. Acad. Sci. USA.

[69] Gorges T.M., Tinhofer I., Drosch M., Röse L., Zollner T.M., Krahn T., von Ahsen O. (2012). Circulating tumour cells escape from EpCAM-based detection due to epithelial-to-mesenchymal transition. BMC Cancer.

[70] Liu Z., Fusi A., Klopocki E., Schmittel A., Tinhofer I., Nonnenmacher A., Keilholz U. (2011). Negative enrichment by immunomagnetic nanobeads for unbiased characterization of circulating tumor cells from peripheral blood of cancer patients. J. Transl. Med..

[71] Lapin M., Tjensvoll K., Oltedal S., Buhl T., Gilje B., Smaaland R., Nordgård O. (2016). MINDEC-An enhanced negative depletion strategy for circulating tumour cell enrichment. Sci. Rep..

[72] Hillig T., Horn P., Nygaard A.-B., Haugaard A.S., Nejlund S., Brandslund I., Sölétormos G. (2015). In vitro detection of circulating tumor cells compared by the CytoTrack and CellSearch methods. Tumor Biol..

[73] Somlo G., Lau S.K., Frankel P., Hsieh H.B., Liu X., Yang L., Krivacic R., Bruce R.H. (2011). Multiple biomarker expression on circulating tumor cells in comparison to tumor tissues from primary and metastatic sites in patients with locally advanced/inflammatory, and stage IV breast cancer, using a novel detection technology. Breast Cancer Res. Treat..

[74] Marrinucci D., Bethel K., Kolatkar A., Luttgen M.S., Malchiodi M., Baehring F., Voigt K., Lazar D., Nieva J., Bazhenova L. (2012). Fluid biopsy in patients with metastatic prostate, pancreatic and breast cancers. Phys. Biol..

[75] Arsenic R., Treue D., Lehmann A., Hummel M., Dietel M., Denkert C., Budczies J. (2015). Comparison of targeted next-generation sequencing and Sanger sequencing for the detection of PIK3CA mutations in breast cancer. BMC Clin. Pathol..

[76] Stasik S., Schuster C., Ortlepp C., Platzbecker U., Bornhäuser M., Schetelig J., Ehninger G., Folprecht G., Thiede C. (2018). An optimized targeted Next-Generation Sequencing approach for sensitive detection of single nucleotide variants. Biomol. Detect. Quantif..

[77] Kwan T.T., Bardia A., Spring L.M., Giobbie-Hurder A., Kalinich M., Dubash T., Sundaresan T., Hong X., LiCausi J.A., Ho U. (2018). A digital RNA signature of circulating tumor cells predicting early therapeutic response in localized and metastatic breast cancer. Cancer Discov..

[78] Pantel K., Cote R.J., Fodstad O. (1999). Detection and clinical importance of micrometastatic disease. JNCI J. Natl. Cancer Inst..

[79] Anjanappa M., Hao Y., Simpson E.R., Bhat-Nakshatri P., Nelson J.B., Tersey S.A., Mirmira R.G., Cohen-Gadol A.A., Saadatzadeh M.R., Li L. (2018). A system for detecting high impact-low frequency mutations in primary tumors and metastases. Oncogene.

[80] Yan W.-T., Cui X., Chen Q., Li Y.-F., Cui Y.-H., Wang Y., Jiang J. (2017). Circulating tumor cell status monitors the treatment responses in breast cancer patients: A meta-analysis. Sci. Rep..

[81] Riethdorf S., Müller V., Loibl S., Nekljudova V., Weber K., Huober J., Fehm T., Schrader I., Hilfrich J., Holms F. (2017). Prognostic impact of circulating tumor cells for breast cancer patients treated in the neoadjuvant “geparquattro” trial. Clin. Cancer Res..

[82] Bidard F.-C., Michiels S., Riethdorf S., Mueller V., Esserman L.J., Lucci A., Naume B., Horiguchi J., Gisbert-Criado R., Sleijfer S. (2018). Circulating tumor cells in breast cancer patients treated by neoadjuvant chemotherapy: A meta-analysis. JNCI J. Natl. Cancer Inst..

[83] Lucci A., Hall C.S., Lodhi A.K., Bhattacharyya A., Anderson A.E., Xiao L., Bedrosian I., Kuerer H.M., Krishnamurthy S. (2012). Circulating tumour cells in non-metastatic breast cancer: A prospective study. Lancet Oncol..

[84] Ignatiadis M., Xenidis N., Perraki M., Apostolaki S., Politaki E., Kafousi M., Stathopoulos E.N., Stathopoulou A., Lianidou E., Chlouverakis G. (2007). Different prognostic value of cytokeratin-19 mRNA–positive circulating tumor cells according to estrogen receptor and HER2 status in early-stage breast cancer. J. Clin. Oncol..

[85] Molloy T.J., Bosma A.J., Baumbusch L.O., Synnestvedt M., Borgen E., Russnes H.G., Schlichting E., van’t Veer L.J., Naume B. (2011). The prognostic significance of tumour cell detection in the peripheral blood versus the bone marrow in 733 early-stage breast cancer patients. Breast Cancer Res..

[86] Rack B., Schindlbeck C., Jückstock J., Andergassen U., Hepp P., Zwingers T., Friedl T.W.P., Lorenz R., Tesch H., Fasching P.A. (2014). Circulating tumor cells predict survival in early average-to-high risk breast cancer patients. J. Natl. Cancer Inst..

[87] Lv Q., Gong L., Zhang T., Ye J., Chai L., Ni C., Mao Y. (2016). Prognostic value of circulating tumor cells in metastatic breast cancer: A systemic review and meta-analysis. Clin. Transl. Oncol..

[88] Liu M.C., Shields P.G., Warren R.D., Cohen P., Wilkinson M., Ottaviano Y.L., Rao S.B., Eng-wong J., Seillier-moiseiwitsch F., Noone A. (2009). Circulating tumor cells: A useful predictor of treatment efficacy in metastatic breast cancer. J. Clin. Oncol..

[89] Ma S., Ling F., Gui A., Chen S., Sun Y., Li Z. (2017). Predictive value of circulating tumor cells for evaluating short- and long-term efficacy of chemotherapy for breast cancer. Med. Sci. Monit..

[90] Smerage J.B., Barlow W.E., Hortobagyi G.N., Winer E.P., Leyland-Jones B., Srkalovic G., Tejwani S., Schott A.F., O’Rourke M.A., Lew D.L. (2014). Circulating tumor cells and response to chemotherapy in metastatic breast cancer: SWOG S0500. J. Clin. Oncol..

[91] Kalinsky K., Mayer J.A., Xu X., Pham T., Wong K.L., Villarin E., Pircher T.J., Brown M., Maurer M.A., Bischoff F.Z. (2015). Correlation of hormone receptor status between circulating tumor cells, primary tumor, and metastasis in breast cancer patients. Clin. Transl. Oncol..

[92] Aktas B., Müller V., Tewes M., Zeitz J., Kasimir-Bauer S., Loehberg C.R., Rack B., Schneeweiss A., Fehm T. (2011). Comparison of estrogen and progesterone receptor status of circulating tumor cells and the primary tumor in metastatic breast cancer patients. Gynecol. Oncol..

[93] Babayan A., Hannemann J., Spötter J., Müller V., Pantel K., Joosse S.A. (2013). Heterogeneity of estrogen receptor expression in circulating tumor cells from metastatic breast cancer patients. PLoS ONE.

[94] Bock C., Rack B., Kuhn C., Hofmann S., Finkenzeller C., Jäger B., Jeschke U., Doisneau-Sixou S.F. (2012). Heterogeneity of ERα and ErbB2 status in cell lines and circulating tumor cells of metastatic breast cancer patients. Transl. Oncol..

[95] Sinkala E., Sollier-Christen E., Renier C., Rosàs-Canyelles E., Che J., Heirich K., Duncombe T.A., Vlassakis J., Yamauchi K.A., Huang H. (2017). Profiling protein expression in circulating tumour cells using microfluidic western blotting. Nat. Commun..

[96] Miyamoto D.T. (2016). Single-cell analysis of circulating tumor cells as a window into tumor heterogeneity. Cold Spring Harb. Symp. Quant. Biol..

[97] Kasimir-Bauer S., Hoffmann O., Wallwiener D., Kimmig R., Fehm T. (2012). Expression of stem cell and epithelial-mesenchymal transition markers in primary breast cancer patients with circulating tumor cells. Breast Cancer Res..

[98] Hensler M., Van Curov A I., Becht E., Rej Palata O., Strnad P., Tesa Rov A E P., Cabi Nakov A E M., Svec D., Kubista M., Bartu Nkov A J.R. (2015). Gene expression profiling of circulating tumor cells and peripheral blood mononuclear cells from breast cancer patients. Oncoimmunology.

[99] Aktas B., Tewes M., Fehm T., Hauch S., Kimmig R., Kasimir- S. (2009). Stem cell and epithelial-mesenchymal transition markers are frequently overexpressed in circulating tumor cells of metastatic breast cancer patients. Breast Cancer Res..

[100] Paoletti C., Larios J.M., Muñiz M.C., Aung K., Cannell E.M., Darga E.P., Kidwell K.M., Thomas D.G., Tokudome N., Brown M.E. (2016). Heterogeneous estrogen receptor expression in circulating tumor cells suggests diverse mechanisms of fulvestrant resistance. Mol. Oncol..

[101] Nadal R., Fernandez A., Sanchez-Rovira P., Salido M., Rodríguez M., García-Puche J.L., Macià M., Corominas J.M., Delgado-Rodriguez M., Gonzalez L. (2012). Biomarkers characterization of circulating tumour cells in breast cancer patients. Breast Cancer Res..

[102] Fehm T., Hoffmann O., Aktas B., Becker S., Solomayer E.F., Wallwiener D., Kimmig R., Kasimir-bauer S. (2009). Detection and characterization of circulating tumor cells in blood of primary breast cancer patients by RT-PCR and comparison to status of bone marrow disseminated cells. Br Can. Res..

[103] Fehm T., Müller V., Aktas B., Janni W., Schneeweiss A., Stickeler E., Lattrich C., Löhberg C.R., Solomayer E., Rack B. (2010). HER2 status of circulating tumor cells in patients with metastatic breast cancer: a prospective, multicenter trial. Breast Cancer Res. Treat..

[104] Alix-Panabières C., Pantel K. (2014). Challenges in circulating tumour cell research. Nat. Rev. Cancer.

[105] Beije N., Onstenk W., Kraan J., Sieuwerts A.M., Hamberg P., Dirix L.Y., Brouwer A., de Jongh F.E., Jager A., Seynaeve C.M. (2016). Prognostic impact of HER2 and ER status of circulating tumor cells in metastatic breast cancer patients with a HER2-negative primary tumor. Neoplasia.

[106] Wallwiener M., Hartkopf A.D., Riethdorf S., Nees J., Sprick M.R., Schönfisch B., Taran F.-A., Heil J., Sohn C., Pantel K. (2015). The impact of HER2 phenotype of circulating tumor cells in metastatic breast cancer: A retrospective study in 107 patients. BMC Cancer.

[107] Onstenk W., Sieuwerts A.M., Weekhout M., Mostert B., Reijm E.A., van Deurzen C.H.M., Bolt-de Vries J.B., Peeters D.J., Hamberg P., Seynaeve C. (2015). Gene expression profiles of circulating tumor cells versus primary tumors in metastatic breast cancer. Cancer Lett..

[108] Hayashi N., Nakamura S., Tokuda Y., Shimoda Y., Yagata H., Yoshida A., Ota H., Hortobagyi G.N., Cristofanilli M., Ueno N.T. (2012). Prognostic value of HER2-positive circulating tumor cells in patients with metastatic breast cancer. Int. J. Clin. Oncol..

[109] Wang C.-H., Chang C.-J., Yeh K.-Y., Chang P.-H., Huang J.-S. (2017). The prognostic value of her2-positive circulating tumor cells in breast cancer patients: A systematic review and meta-analysis. Clin. Breast Cancer.

[110] Paoletti C., Muñiz M.C., Thomas D.G., Griffith K.A., Kidwell K.M., Tokudome N., Brown M.E., Aung K., Miller M.C., Blossom D.L. (2015). Development of circulating tumor cell-endocrine therapy index in patients with hormone receptor-positive breast cancer. Clin. Cancer Res..

[111] Cizkova M., Dujaric M.-E., Lehmann-Che J., Scott V., Tembo O., Asselain B., Pierga J.-Y., Marty M., de Cremoux P., Spyratos F. (2013). Outcome impact of PIK3CA mutations in HER2-positive breast cancer patients treated with trastuzumab. Br. J. Cancer.

[112] Polzer B., Medoro G., Pasch S., Fontana F., Zorzino L., Pestka A., Andergassen U., Meier-Stiegen F., Czyz Z.T., Alberter B. (2014). Molecular profiling of single circulating tumor cells with diagnostic intention. EMBO Mol. Med..

[113] Punnoose E.A., Atwal S.K., Spoerke J.M., Savage H., Pandita A., Yeh R.-F., Pirzkall A., Fine B.M., Amler L.C., Chen D.S. (2010). Molecular biomarker analyses using circulating tumor cells. PLoS ONE.

[114] Frithiof H., Aaltonen K., Rydén L. (2016). A FISH-based method for assessment of HER-2 amplification status in breast cancer circulating tumor cells following CellSearch isolation. Onco. Targets. Ther..

[115] Mayer J.A., Pham T., Wong K.L., Scoggin J., Sales E.V., Clarin T., Pircher T.J., Mikolajczyk S.D., Cotter P.D., Bischoff F.Z. (2011). FISH-based determination of HER2 status in circulating tumor cells isolated with the microfluidic CEE^TM^ platform. Cancer Genet..

[116] Bidard F.-C., Cottu P., Dubot C., Venat-Bouvet L., Lortholary A., Bourgeois H., Bollet M., Servent Hanon V., Luporsi-Gely E., Espie M. (2017). 117PAnti-HER2 therapy efficacy in HER2-negative metastatic breast cancer with HER2-amplified circulating tumor cells: Results of the CirCe T-DM1 trial. Ann. Oncol..

[117] Neves R.P.L., Raba K., Schmidt O., Honisch E., Meier-Stiegen F., Behrens B., Möhlendick B., Fehm T., Neubauer H., Klein C.A. (2014). Genomic high-resolution profiling of single CKpos/CD45neg flow-sorting purified circulating tumor cells from patients with metastatic breast cancer. Clin. Chem..

[118] Bingham C., Fernandez S.V., Fittipaldi P., Dempsey P.W., Ruth K.J., Cristofanilli M., Katherine Alpaugh R. (2017). Mutational studies on single circulating tumor cells isolated from the blood of inflammatory breast cancer patients. Breast Cancer Res. Treat..

[119] Mu Z., Benali-Furet N., Uzan G., Znaty A., Ye Z., Paolillo C., Wang C., Austin L., Rossi G., Fortina P. (2016). Detection and characterization of circulating tumor associated cells in metastatic breast cancer. Int. J. Mol. Sci..

[120] De Luca F., Rotunno G., Salvianti F., Galardi F., Pestrin M., Gabellini S., Simi L., Mancini I., Vannucchi A.M., Pazzagli M. (2016). Mutational analysis of single circulating tumor cells by next generation sequencing in metastatic breast cancer. Oncotarget.

[121] Apostolaki S., Perraki M., Kallergi G., Kafousi M., Papadopoulos S., Kotsakis A., Pallis A., Xenidis N., Kalmanti L., Kalbakis K. (2009). Detection of occult HER2 mRNA-positive tumor cells in the peripheral blood of patients with operable breast cancer: Evaluation of their prognostic relevance. Breast Cancer Res. Treat..

[122] Ignatiadis M., Kallergi G., Ntoulia M., Perraki M., Apostolaki S., Kafousi M., Chlouverakis G., Stathopoulos E., Lianidou E., Georgoulias V. (2008). Prognostic value of the molecular detection of circulating tumor cells using a multimarker reverse transcription-pcr assay for cytokeratin 19, mammaglobin a, and her2 in early breast cancer. Clin. Cancer Res..

[123] Ignatiadis M., Rothé F., Chaboteaux C., Durbecq V., Rouas G., Criscitiello C., Metallo J., Kheddoumi N., Singhal S.K., Michiels S. (2011). HER2-positive circulating tumor cells in breast cancer. PLoS ONE.

[124] Markiewicz A., Ahrends T., Wełnicka-Jaśkiewicz M., Seroczyńska B., Skokowski J., Jaśkiewicz J., Szade J., Biernat W., Zaczek A.J. (2012). Expression of epithelial to mesenchymal transition-related markers in lymph node metastases as a surrogate for primary tumor metastatic potential in breast cancer. J. Transl. Med..

[125] Sethi S., Sarkar F.H., Ahmed Q., Bandyopadhyay S., Nahleh Z.A., Semaan A., Sakr W., Munkarah A., Ali-Fehmi R. (2011). Molecular markers of epithelial-to-mesenchymal transition are associated with tumor aggressiveness in breast carcinoma. Transl. Oncol..

[126] Lang J.E., Scott J.H., Wolf D.M., Novak P., Punj V., Magbanua M.J.M., Zhu W., Mineyev N., Haqq C.M., Crothers J.R. (2015). Expression profiling of circulating tumor cells in metastatic breast cancer. Breast Cancer Res. Treat..

[127] Porras T.B., Kaur P., Ring A., Schechter N., Lang J.E. (2018). Challenges in using liquid biopsies for gene expression profiling. Oncotarget.

[128] Sieuwerts A.M., Mostert B., Bolt-De Vries J., Peeters D., De Jongh F.E., Stouthard J.M.L., Dirix L.Y., Van Dam P.A., Van Galen A., De Weerd V. (2011). mRNA and microRNA expression profiles in circulating tumor cells and primary tumors of metastatic breast cancer patients. Clin. Cancer Res..

[129] Fina E., Callari M., Reduzzi C., D’Aiuto F., Mariani G., Generali D., Pierotti M.A., Daidone M.G., Cappelletti V. (2015). Gene expression profiling of circulating tumor cells in breast cancer. Clin. Chem..

[130] Aaltonen K.E., Novosadová V., Bendahl P., Graffman C., Larsson A., Rydén L. (2017). Molecular characterization of circulating tumor cells from patients with metastatic breast cancer reflects evolutionary changes in gene expression under the pressure of systemic therapy Patient cohort. Oncotarget.

[131] Leong S.M., Tan K.M.-L., Chua H.W., Huang M.-C., Cheong W.C., Li M.-H., Tucker S., Koay E.S.-C. (2017). Paper-based MicroRNA expression profiling from plasma and circulating tumor cells. Clin. Chem..

[132] Gasch C., Plummer P.N., Jovanovic L., McInnes L.M., Wescott D., Saunders C.M., Schneeweiss A., Wallwiener M., Nelson C., Spring K.J. (2015). Heterogeneity of miR-10b expression in circulating tumor cells. Sci. Rep..

[133] Tellez-Gabriel M., Ory B., Lamoureux F., Heymann M.-F., Heymann D., Tellez-Gabriel M., Ory B., Lamoureux F., Heymann M.-F., Heymann D. (2016). Tumour heterogeneity: The key advantages of single-cell analysis. Int. J. Mol. Sci..

[134] Soliman N.A., Yussif S.M. (2016). Ki-67 as a prognostic marker according to breast cancer molecular subtype. Cancer Biol. Med..

[135] Niikura N., Masuda S., Kumaki N., Xiaoyan T., Terada M., Terao M., Iwamoto T., Oshitanai R., Morioka T., Tuda B. (2014). Prognostic significance of the Ki67 scoring categories in breast cancer subgroups. Clin. Breast Cancer.

[136] Gould Rothberg B.E., Bracken M.B. (2006). E-cadherin immunohistochemical expression as a prognostic factor in infiltrating ductal carcinoma of the breast: A systematic review and meta-analysis. Breast Cancer Res. Treat..

[137] Thomas P.A., Kirschmann D.A., Cerhan J.R., Folberg R., Seftor E.A., Sellers T.A., Hendrix M.J. (1999). Association between keratin and vimentin expression, malignant phenotype, and survival in postmenopausal breast cancer patients. Clin. Cancer Res..

[138] Bulfoni M., Gerratana L., Del Ben F., Marzinotto S., Sorrentino M., Turetta M., Scoles G., Toffoletto B., Isola M., Beltrami C.A. (2016). In patients with metastatic breast cancer the identification of circulating tumor cells in epithelial-to-mesenchymal transition is associated with a poor prognosis. Breast Cancer Res..

[139] Gonzalez-Angulo A.M., Hennessy B.T., Meric-Bernstam F., Sahin A., Liu W., Ju Z., Carey M.S., Myhre S., Speers C., Deng L. (2011). Functional proteomics can define prognosis and predict pathologic complete response in patients with breast cancer. Clin. Proteomics.

[140] Gonzalez-Angulo A.M., Liu S., Chen H., Chavez-MacGregor M., Sahin A., Hortobagyi G.N., Mills G.B., Do K.-A., Meric-Bernstam F. (2013). Functional proteomics characterization of residual breast cancer after neoadjuvant systemic chemotherapy. Ann. Oncol..

[141] Macaulay I.C., Voet T. (2014). Single cell genomics: Advances and future perspectives. PLoS Genet..

[142] Fernandez S.V., Bingham C., Fittipaldi P., Austin L., Palazzo J., Palmer G., Alpaugh K., Cristofanilli M. (2014). TP53 mutations detected in circulating tumor cells present in the blood of metastatic triple negative breast cancer patients. Breast Cancer Res..

[143] Pestrin M., Salvianti F., Galardi F., De Luca F., Turner N., Malorni L., Pazzagli M., Di Leo A., Pinzani P. (2015). Heterogeneity of *PIK3CA* mutational status at the single cell level in circulating tumor cells from metastatic breast cancer patients. Mol. Oncol..

[144] Deng G., Krishnakumar S., Powell A.A., Zhang H., Mindrinos M.N., Telli M.L., Davis R.W., Jeffrey S.S. (2014). Single cell mutational analysis of PIK3CA in circulating tumor cells and metastases in breast cancer reveals heterogeneity, discordance, and mutation persistence in cultured disseminated tumor cells from bone marrow. BMC Cancer.

[145] Flores L.M., Kindelberger D.W., Ligon A.H., Capelletti M., Fiorentino M., Loda M., Cibas E.S., Jänne P.A., Krop I.E. (2010). Improving the yield of circulating tumour cells facilitates molecular characterisation and recognition of discordant HER2 amplification in breast cancer. Br. J. Cancer.

[146] Paoletti C., Cani A.K., Larios J.M., Hovelson D.H., Aung K., Darga E.P., Cannell E.M., Baratta P.J., Liu C.-J., Chu D. (2018). Comprehensive mutation and copy number profiling in archived circulating breast cancer tumor cells documents heterogeneous resistance mechanisms. Cancer Res..

[147] Berns K., Horlings H.M., Hennessy B.T., Madiredjo M., Hijmans E.M., Beelen K., Linn S.C., Gonzalez-Angulo A.M., Stemke-Hale K., Hauptmann M. (2007). A functional genetic approach identifies the PI3K pathway as a major determinant of trastuzumab resistance in breast cancer. Cancer Cell.

[148] Chandarlapaty S., Sakr R.A., Giri D., Patil S., Heguy A., Morrow M., Modi S., Norton L., Rosen N., Hudis C. (2012). Frequent mutational activation of the PI3K-AKT pathway in trastuzumab-resistant breast cancer. Clin. Cancer Res..

[149] Li J., Yang L., Gaur S., Zhang K., Wu X., Yuan Y.-C., Li H., Hu S., Weng Y., Yen Y. (2014). Mutants TP53 p.R273H and p.R273C but not p.R273G enhance cancer cell malignancy. Hum. Mutat..

[150] Bose R., Kavuri S.M., Searleman A.C., Shen W., Shen D., Koboldt D.C., Monsey J., Goel N., Aronson A.B., Li S. (2013). Activating HER2 mutations in HER2 gene amplification negative breast cancer. Cancer Discov..

[151] Pereira C.B.L., Leal M.F., de Souza C.R.T., Montenegro R.C., Rey J.A., Carvalho A.A., Assumpção P.P., Khayat A.S., Pinto G.R., Demachki S. (2013). Prognostic and predictive significance of myc and kras alterations in breast cancer from women treated with neoadjuvant chemotherapy. PLoS ONE.

[152] Aas T., Børresen A.-L., Geisler S., Smith-Sørensen B., Johnsen H., Varhaug J.E., Akslen L.A., Lønning P.E. (1996). Specific P53 mutations are associated with de novo resistance to doxorubicin in breast cancer patients. Nat. Med..

[153] Berns E.M., Foekens J.A., Vossen R., Look M.P., Devilee P., Henzen-Logmans S.C., van Staveren I.L., van Putten W.L., Inganäs M., Meijer-van Gelder M.E. (2000). Complete sequencing of TP53 predicts poor response to systemic therapy of advanced breast cancer. Cancer Res..

[154] Kandioler-Eckersberger D., Ludwig C., Rudas M., Kappel S., Janschek E., Wenzel C., Schlagbauer-Wadl H., Mittlböck M., Gnant M., Steger G. (2000). TP53 mutation and p53 overexpression for prediction of response to neoadjuvant treatment in breast cancer patients. Clin. Cancer Res..

[155] Muller P.A.J., Vousden K.H. (2014). Mutant p53 in cancer: New functions and therapeutic opportunities. Cancer Cell.

[156] Lang J.E., Magbanua M.J.M., Scott J.H., Makrigiorgos G.M., Wang G., Federman S., Esserman L.J., Park J.W., Haqq C.M. (2009). A comparison of RNA amplification techniques at sub-nanogram input concentration. BMC Genomics.

[157] Fehm T., Becker S., Duerr-Stoerzer S., Sotlar K., Mueller V., Wallwiener D., Lane N., Solomayer E., Uhr J. (2007). Determination of HER2 status using both serum HER2 levels and circulating tumor cells in patients with recurrent breast cancer whose primary tumor was HER2 negative or of unknown HER2 status. Breast Cancer Res..

[158] Tewes M., Aktas B., Welt A., Mueller S., Hauch S., Kimmig R., Kasimir-Bauer S. (2009). Molecular profiling and predictive value of circulating tumor cells in patients with metastatic breast cancer: An option for monitoring response to breast cancer related therapies. Breast Cancer Res. Treat..

[159] Guan X., Ma F., Liu S., Wu S., Xiao R., Yuan L., Sun X., Yi Z., Yang H., Xu B. (2016). Analysis of the hormone receptor status of circulating tumor cell subpopulations based on epithelial-mesenchymal transition: A proof-of-principle study on the heterogeneity of circulating tumor cells. Oncotarget.

[160] Lamouille S., Xu J., Derynck R. (2014). Molecular mechanisms of epithelial-mesenchymal transition. Nat. Rev. Mol. Cell Biol..

[161] Yilmaz M., Christofori G. (2009). EMT, the cytoskeleton, and cancer cell invasion. Cancer Metastasis Rev..

[162] Thiery J.P., Acloque H., Huang R.Y.J., Nieto M.A. (2009). Epithelial-mesenchymal transitions in development and disease. Cell.

[163] Gradilone A., Raimondi C., Nicolazzo C., Petracca A., Gandini O., Vincenzi B., Naso G., Aglianò A.M., Cortesi E., Gazzaniga P. (2011). Circulating tumour cells lacking cytokeratin in breast cancer: The importance of being mesenchymal. J. Cell. Mol. Med..

[164] Theodoropoulos P.A., Polioudaki H., Agelaki S., Kallergi G., Saridaki Z., Mavroudis D., Georgoulias V. (2010). Circulating tumor cells with a putative stem cell phenotype in peripheral blood of patients with breast cancer. Cancer Lett..

[165] Singh A., Settleman J. (2010). EMT, cancer stem cells and drug resistance: An emerging axis of evil in the war on cancer. Oncogene.

[166] Seton-Rogers S. (2016). Epithelial-mesenchymal transition: Untangling EMT’s functions. Nat. Rev. Cancer.

[167] Armstrong A.J., Marengo M.S., Oltean S., Kemeny G., Bitting R.L., Turnbull J.D., Herold C.I., Marcom P.K., George D.J., Garcia-Blanco M.A. (2011). Circulating tumor cells from patients with advanced prostate and breast cancer display both epithelial and mesenchymal markers. Mol. Cancer Res..

[168] Karagiannis G.S., Goswami S., Jones J.G., Oktay M.H., Condeelis J.S. (2016). Signatures of breast cancer metastasis at a glance. J. Cell Sci..

[169] Bredemeier M., Edimiris P., Tewes M., Mach P., Aktas B., Schellbach D., Wagner J., Kimmig R., Kasimir-Bauer S. (2016). Establishment of a multimarker qPCR panel for the molecular characterization of circulating tumor cells in blood samples of metastatic breast cancer patients during the course of palliative treatment. Oncotarget.

[170] Boral D., Vishnoi M., Liu H.N., Yin W., Sprouse M.L., Scamardo A., Hong D.S., Tan T.Z., Thiery J.P., Chang J.C. (2017). Molecular characterization of breast cancer CTCs associated with brain metastasis. Nat. Commun..

[171] Patsialou A., Wang Y., Lin J., Whitney K., Goswami S., Kenny P.A., Condeelis J.S. (2012). Selective gene-expression profiling of migratory tumor cells in vivo predicts clinical outcome in breast cancer patients. Breast Cancer Res..

[172] Molloy T.J., Roepman P., Naume B., van’t Veer L.J. (2012). A prognostic gene expression profile that predicts circulating tumor cell presence in breast cancer patients. PLoS ONE.

[173] Curtis C. (2015). Genomic profiling of breast cancers. Curr. Opin. Obstet. Gynecol..

[174] Gorges T.M., Kuske A., Röck K., Mauermann O., Müller V., Peine S., Verpoort K., Novosadova V., Kubista M., Riethdorf S. (2016). Accession of tumor heterogeneity by multiplex transcriptome profiling of single circulating tumor cells. Clin. Chem..

[175] Navin N., Kendall J., Troge J., Andrews P., Rodgers L., McIndoo J., Cook K., Stepansky A., Levy D., Esposito D. (2011). Tumour evolution inferred by single-cell sequencing. Nature.

[176] Powell A.A., Talasaz A.H., Zhang H., Coram M.A., Reddy A., Deng G., Telli M.L., Advani R.H., Carlson R.W., Mollick J.A. (2012). Single cell profiling of circulating tumor cells: Transcriptional heterogeneity and diversity from breast cancer cell lines. PLoS ONE.

[177] Dey S.S., Kester L., Spanjaard B., Bienko M., van Oudenaarden A. (2015). Integrated genome and transcriptome sequencing of the same cell. Nat. Biotechnol..

[178] Macaulay I.C., Haerty W., Kumar P., Li Y.I., Hu T.X., Teng M.J., Goolam M., Saurat N., Coupland P., Shirley L.M. (2015). G&T-seq: Parallel sequencing of single-cell genomes and transcriptomes. Nat. Methods.

[179] Genshaft A.S., Li S., Gallant C.J., Darmanis S., Prakadan S.M., Ziegler C.G.K., Lundberg M., Fredriksson S., Hong J., Regev A. (2016). Multiplexed, targeted profiling of single-cell proteomes and transcriptomes in a single reaction. Genome Biol..

[180] Gulbahce N., Magbanua M.J.M., Chin R., Agarwal M.R., Luo X., Liu J., Hayden D.M., Mao Q., Ciotlos S., Li Z. (2017). Quantitative whole genome sequencing of circulating tumor cells enables personalized combination therapy of metastatic cancer. Cancer Res..

[181] Ilicic T., Kim J.K., Kolodziejczyk A.A., Bagger F.O., McCarthy D.J., Marioni J.C., Teichmann S.A. (2016). Classification of low quality cells from single-cell RNA-seq data. Genome Biol..

[182] Kumar N., Singh A., Kulkarni R.V. (2015). Transcriptional bursting in gene expression: Analytical results for general stochastic models. PLOS Comput. Biol..

[183] Tyagi S. (2015). Tuning noise in gene expression. Mol. Syst. Biol..

[184] Ziegenhain C., Vieth B., Parekh S., Heyn H. (2017). Comparative analysis of single-cell RNA sequencing methods. Mol. Cell.

